# Impact of Generic Price Linkage System and Reference Price System on prices of pharmaceuticals – comparison of Austria and Finland

**DOI:** 10.1186/2052-3211-8-S1-P2

**Published:** 2015-10-05

**Authors:** Jaana E Martikainen, Timo Maljanen, Hanna Koskinen, Sabine Vogler

**Affiliations:** 1 Social Insurance Institution, Research Department, Helsinki, 101, Finland; 2 WHO Collaborating Centre for Pricing and Reimbursement Policies, Department of Health Economics, Gesundheit Österreich GmbH (Austrian Public Health Institute), Vienna, 1010, Austria

## Background

To contain costs of medicines, many countries have introduced policies aiming to lower prices of generics[1]. Measures taken in Austria include Generic Price Linkage and in Finland Generic Substitution and Reference Pricing, where competition plays a crucial role.

## Objectives

The aim of this study is to assess the Generic Price Linkage system and the system that includes Generic Substitution and Reference Pricing by comparing prices of generics and originators and the number of generics entering the market in Austria and in Finland.

Policies targeted: Generic Price Linkage, Generic Substitution, Reference Pricing.

Stakeholders: Austria: Main Association of Austrian Social Security Institutions, pharmaceutical industry, patients. Finland: Finnish Medicines Agency, Pharmaceuticals Pricing Board, Social Insurance Institution, pharmaceutical industry, pharmacies, patients.

Region covered: EURO, Austria and Finland.

## Methods

Study design: Policy evaluation. Time series design was used to estimate changes in price levels.

Time period: 2009–2013.

Setting: Pharmaceuticals used in outpatient care and prescribed either in the public or private sector. Ten active ingredients with high sales in Finland and reimbursable in both countries were included in the analysis.

Interventions: Prices of original products whose patent protection expired during 2010–2012 and generics comparable with them were analysed 6 months before and 12 months after generic entry. Price levels were measured in wholesale prices proportioned to the number of Defined Daily Doses in the package (EUR/DDD).

## Results

One year after generic entry, prices of the originators had fallen, on average, by 46% in Austria and by 21% in Finland. Prices of the generics were 66% lower in Austria and 59% lower in Finland than prices of the originators before generic entry. The mean number of generics per active ingredient was 6.3 in Austria and 5.1 in Finland.

## Conclusions and lessons learned

Even if uptake of generics is lower in Austria (26% in volume) than in Finland (36%), the Austrian pricing system appears to be more efficient to lower prices. Price competition in Finland is probably reduced by a concentrated generic market.

It has been stated that free competition lowers generic prices more efficiently than linking the price of a generic to the price of the originator[2]. That is not necessarily the case. Success of a policy measure also largely depends on how the details of the measure are constructed.
